# Airborne gamma-ray mapping using fixed-wing vertical take-off and landing (VTOL) uncrewed aerial vehicles

**DOI:** 10.3389/frobt.2023.1137763

**Published:** 2023-06-28

**Authors:** Ewan Woodbridge, Dean T. Connor, Yannick Verbelen, Duncan Hine, Tom Richardson, Thomas B. Scott

**Affiliations:** ^1^ H. H. Wills Physics Laboratory, Interface Analysis Centre, University of Bristol, Bristol, United Kingdom; ^2^ National Nuclear Laboratory, Warrington, United Kingdom; ^3^ Bristol Flight Lab, Faculty of Engineering, University of Bristol, University Walk, Bristol, United Kingdom

**Keywords:** VTOL (vertical takeoff and landing), radiation mapping, UAV (unmanned aerial vehicle), naturally occurring radioactive material (NORM), radioactivity, gamma radiation

## Abstract

Low-cost uncrewed aerial vehicles (UAVs) are replacing manned aircraft for airborne radiation mapping applications such as nuclear accident response scenarios or surveying ore deposits and mine sites because of their cost-effectiveness and ability to conduct surveys at lower altitude compared to manned counterparts. Both multi-rotor UAVs and fixed-wing UAVs are well established technologies for aerial radiation mapping applications, however, both also have drawbacks: multi-rotor UAVs are very limited in flight time and range, and fixed-wing UAVs usually require facilities for take-off and landing. A compromise solution is introduced in this work, using a fixed-wing vertical take-off and landing (VTOL) UAV that combines the flexibility of a multi-rotor UAV with the range and flight time of a fixed-wing UAV. The first implementation of a VTOL with radiation mapping capabilities is presented, based on a commercial WingtraOne UAV augmented with CsI scintillator and CZT semiconductor gamma spectrometers. The radiation mapping capabilities of the prototype are demonstrated in a case study, mapping the distribution of radionuclides around the South Terras legacy uranium mine in the south of England, United Kingdom, and the results are compared with previous studies using multi-rotor and manned aircraft to survey the same area.

## 1 Introduction

Uncrewed aerial vehicles (UAVs) augmented with radiation mapping technology enable the distribution and variation of intensity of radiation to be mapped around nuclear sites without the need to take any human operators into zones of unknown or high radiation dose (Cai et al., 2016; Connor et al., 2016; 2020; Lowdon et al., 2019; Martin et al., 2020). UAVs have the added benefit of costing a fraction of a full-sized commercial aircraft, be that helicopters or aeroplanes (Han et al., 2013). This fractional cost is not only seen in the purchase price but also in terms of the cost of the infrastructure and expertise needed to maintain the aerial capability and make regular deployments.

One substantial difference between manned aircraft and UAVs, apart from the cost difference, is the relative mass difference. An aeroplane or helicopter crashing into the side of a nuclear facility or any other large piece of civil infrastructure is likely to be terminalfor the aircraft and the aircrew, as well as highly damaging to the building. In comparison, small UAVs weighing under 25 kg would only have a relatively limited kinetic effect on the building even if flying at their highest speed.

UAVs also have substantial flexibility in their deployment *versus* conventional manned aircraft. This is because they do not require, in many cases, an airfield or other large clear take-off area to be deployed and then safely landed. This is best exemplified by multi-rotor drones which can be easily transported by road and deployed into a mission within minutes of arriving at the designated take-off area. Helicopters and planes are limited to sortieing out from airfields where there is specialist infrastructure for refuelling and undertaking necessary mechanical support and repair works.

It is notable that UAVs are not superior to manned aircraft on all counts. One strategically important limitation to recognise is that typical UAVs have a much more limited flight time and range than occupied aircraft due to their reduced size and fuelling capacity. This is especially the case for multi-rotor aircraft and is somewhat exacerbated by the fact that as any sensory payload gets heavier and larger, the respective flight time of the UAVs carrying it will be reduced. This means that for very heavy sensor payloads the flight time of some multi-rotor aircraft is likely to be less than 20 min and dropping to less than 10 min for some of the older UAVs with more limited battery capacity.

Due to the range limitation exhibited by some UAVs, it is currently best considered that radiation mapping UAV technology remains highly complementary to existing manned capabilities and could even represent a partial capability replacement in some instances. As battery technologies and even hydrogen fuel cell technologies continue to advance, it is realistic to expect that the mission capabilities of drones in terms of flight times and therefore range, will continue to improve substantially. Ultimately, within the next 5–10 years it is likely that UAVs will be able to replace a number of existing manned radiological aerial response capabilities across the world.

There is a need for more in-depth consideration as to what types of UAVs are appropriate for different deployment scenarios, as it is not simply a case of one type that works for all scenarios. One important use-case considered is an emergency response scenario. When an unknown amount and type of radioactivity has been released, and radiological materials have escaped confinement, there is a need to immediately gain strategic and tactical insight as to the nature of the release event and the inventory of radioactivity released. In this early initial response phase, when the potential hazard and intensity of the radiation are not well established, it is most desirable from an operational perspective to keep any human operators e.g., pilots, away from the incident site and ideally as far away as possible and in an upwind direction.

Should the area be too large to survey with a multi-rotor UAV, fixed-wing UAVs may be considered a more useful operational tool. There are two discriminating features that make a fixed-wing capability better predisposed for a long-range emergency response mission. Firstly, a fixed-wing drone has a longer flight time, and therefore greater operational range than a multi-rotor counterpart with comparable payload capacity, mainly because it is assisted by the aerodynamic benefit of having wings for the generation of lift, leading to a more efficient mode of flight than multi-rotor systems. Large mono-rotor UAVs are available as an alternative but are substantially more expensive and far more complicated to operate. The second strategic benefit is that fixed-wing systems are much faster than their multi-rotor counterparts and therefore able to cover a much larger area more rapidly. This is a very important capability for emergency response because delineating the risk area is a strategically important early priority. Commercial off-the-shelf (COTS) fixed-wing UAV systems offer flight times upwards of an hour, flying at speeds in excess of 40 km/h (Tilon et al., 2022). Usually, the larger the UAV, the longer the flight time and also the greater the carrying capacity.

Whilst small fixed-wing drones can be launched either by hand or via catapult systems, it is usually the case that the larger the drone the greater the likelihood of the system requiring an airport or heliport for take-off and landing. For fixed-wing drones, the riskiest part of any mission is take-off and landing, with the latter often carrying a higher likelihood of physical damage to the aircraft and its attached payload.

A novel solution in terms of retaining the mission capability of a fixed-wing UAV but without the need for a large take-off and landing area, is the vertical take-off and landing (VTOL) fixed-wing UAV. Such systems represent a technical compromise in terms of slightly reduced flight times *versus* similarly sized fixed-wing counterparts, but still enable rapid capture of radiometric data over multi-kilometre areas much more quickly than a multi-rotor drone. However until very recently, a radiation mapping fixed-wing VTOL capability has not existed. By covering 15× the area in half the time of previous studies that used multi-rotor UAVs, the presented work is to the authors’ knowledge the first experimental report of the use of such a system for mapping radiation normally. This paper validates the concept of using fixed-wing systems with VTOL capability for nuclear emergency response applications and indicates that such systems have excellent utility in nuclear emergency response applications where area mapping is urgently required. This capability might be equally useful in the fields of mineral prospecting and mine site surveying.

## 2 Materials and methods

Table 1 shows a comparison of COTS fixed-wing VTOL UAVs. The WingtraOne Gen One, with a total aircraft takeoff weight of 4.5 kg was selected as the VTOL of choice for its flight time of up to an hour utilising batteries which are just less than the aeroplane safe carry-on limit set by the ICAO (2021) of 100 W h and an interchangeable payload of up to about ≈800 g (Trpković et al., 2020, p. 6).

**TABLE 1:** Comparison of varying commercial off the shelf (COTS) fixed-wing VTOL UAVs based on their nameplate specifications. Simon et al. (2021); Tilon et al. (2022); Simpson et al. (2021); Türk et al. (2022); Ramsankaran et al. (2021).

Fixed-wing VTOL	Flight time	Payload	Battery	Wind resistance	Cruise speed	Cost	Range
Unit	min	g	Wh	km/h	km/h	k£	km
Wingtra one	55	800	99	45	60	18	10
Delta quad	120	1,200	387	43	100	18	10
Trinity F90+	90	500	259	43	61	19	7
ATMOS marlyn	50	1,000	100	45	64	15	7
EBEE X	60	200	75	45	90	19	8

### 2.1 Wingtra-AARM system

**FIGURE 1:**
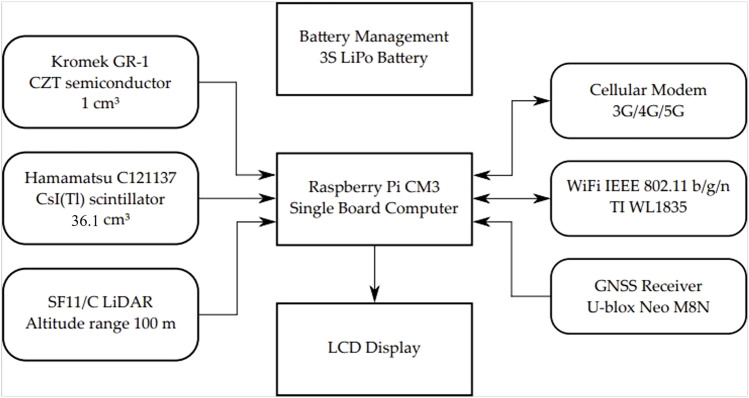
Block diagram of the radiation mapping payload, featuring a 1 cm^3^ CZT semiconductor detector, and a 36.1 cm^3^ CsI(Tl) scintillator detector.

Figure 1 shows a summary block diagram of the bespoke payload designed by Imitec Ltd., For implementation within the WingtraOne Gen One fixed-wing VTOL UAV. The AARM (autonomous airborne radiation mapping) system is designed to work independently of the VTOL with the use of a Hamamatsu C12137-01 CsI(Tl) scintillator (Lowdon et al., 2019) and Kromek GR-1 CZT semiconductor detector in order to add gamma spectrometry radiation mapping capabilities to the UAV. The radiological data is stored and transmitted straight back to the pilot via WIFI connection with a max range of 100 m and over a cellular connection beyond that range to produce live radiation data.

**FIGURE 2:**
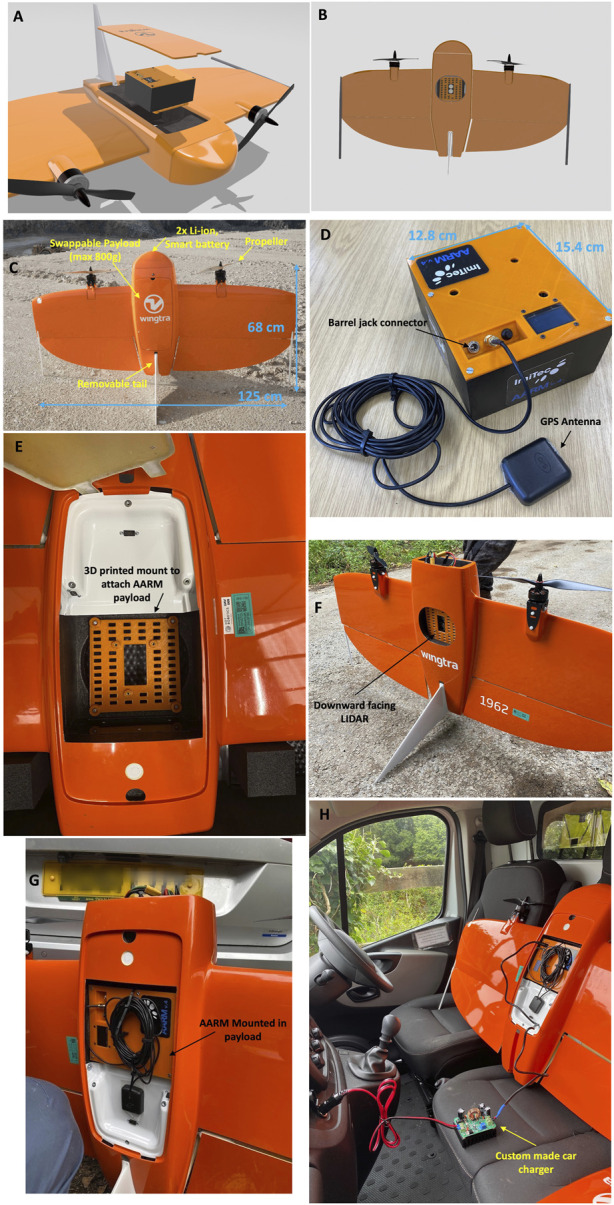
Figures showing the Wingtra-AARM system. **(A,B)** show CAD designs of the AARM within the Wingtra payload. **(C)** shows the main features of the Wingtra one Gen one fixed-wing VTOL. **(D)** shows the AARMv4 detector unit that mounts onto the payload plate seen in **(E–G)**. **(H)** shows the AARM unit charging from a ground vehicle in the field.

Figures 2A–H show the integrated Wingtra-AARM system. The AARM unit replaces the stock Sony RX1 DSLR camera that is provided as standard with the Wingtra for photogrammetry purposes. As Figure 2E shows, the AARM unit was securely mounted within the Wingtra payload bay with the use of a custom plastic mounting plate allowing the AARM unit to remain stationary and as such, not subjecting the unit to vibrations that can affect the performance of the detectors. This is particularly important for the CZT GR1 which has been found to produce noisy data in the presence of high-frequency vibrations (Connor et al., 2020). The crocodile clamps that come with Wingtra’s stock battery station kit allow the UAV batteries to be charged from a car battery. Figure 2H shows the utilisation of car charging capabilities for the AARM unit, meaning that the entire Wingtra-AARM system can be charged from a car, in the field, without the need for an external diesel generator.

### 2.2 Wingtra app and take-off procedures

**FIGURE 3:**
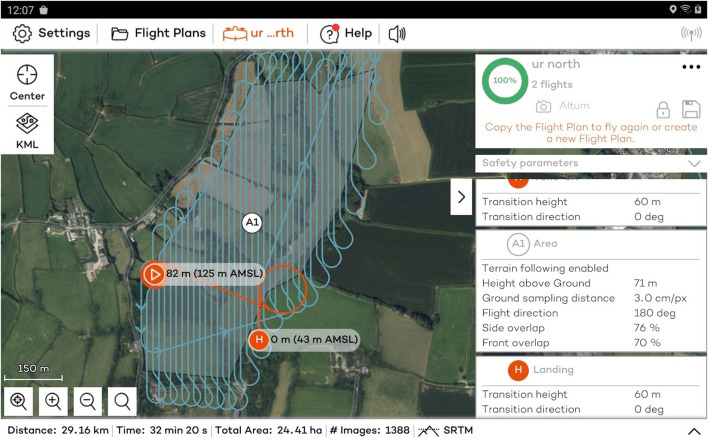
Example flight plan for the South Terras site that is pre-determined by the pilot using the Wingtra Pilot app.

One of the inherent benefits of selecting Wingtra as the VTOL system of choice is the level of flight automation that is provided. The flight is pre-determined by the pilot with the use of the Wingtra pilot app installed on the tablet provided with the UAV. An example of a flight plan used during the South Terras survey can be seen in Figure 3. The only input needed from the pilot is to complete the pre-flight take-off checklist that is prompted by the app upon startup. Automated mission planning will require the pilot to define in the software the area of the flight, the flight line overlap, that is the distance between the flight lines and the altitude the survey is conducted, which is capped at a minimum of 70 m height above ground. Once the pre-flight take-off checklist is completed, the pilot simply activates the mission and the Wingtra undertakes its survey fully automated with no input from the pilot.

As the Wingtra’s commercially intended purpose is photogrammetry, the onboard electronics on the UAV expect to receive input from the DSLR camera during take-off and in flight. This meant that an additional small interface circuit board provided by Wingtra was required to enable take-off without the stock camera payload.

### 2.3 Airborne data acquisition

**FIGURE 4:**
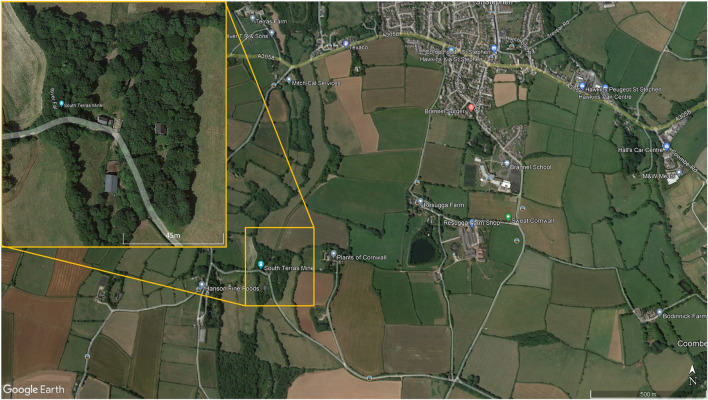
Location of the South Terras legacy uranium mine and refinery site situated 1.5 km South-West of the village of St Stephen in Cornwall.

South Terras is a legacy uranium mine site including a small radium refinery situated 1.5 km south-west of the village of St Stephen, Cornwall, United Kingdom as seen in Figure 4. The site is historically known for its large uranium bearing ore lode, which contains the minerals torbernite (Cu(UO_2_)_2_(PO_4_)_2_ ⋅ 8–12 H_2_O), autunite (Ca(UO_2_)_2_(PO_4_)_2_ ⋅ 10–12 H_2_O) and uraninite (pitchblende, UO_2_), with vein lodes up to 457 m long (Smale, 1993). Radium produced by ore processing was reportedly utilised by Marie Curie in her Nobel prize winning research (Smale, 1993). Due to the mining and radium refining that operated from 1870 to 1930, the area has a notable radioactivity well above the average natural background, with activity ranging from 5 μSv h^−1^ to > 20 µSv h^−1^ at certain locations around the site (Kutner et al., 2012; Martin et al., 2015). This made it an ideal candidate site for deploying and testing the radiation detection capabilities of the fixed-wing VTOL system.

**FIGURE 5:**
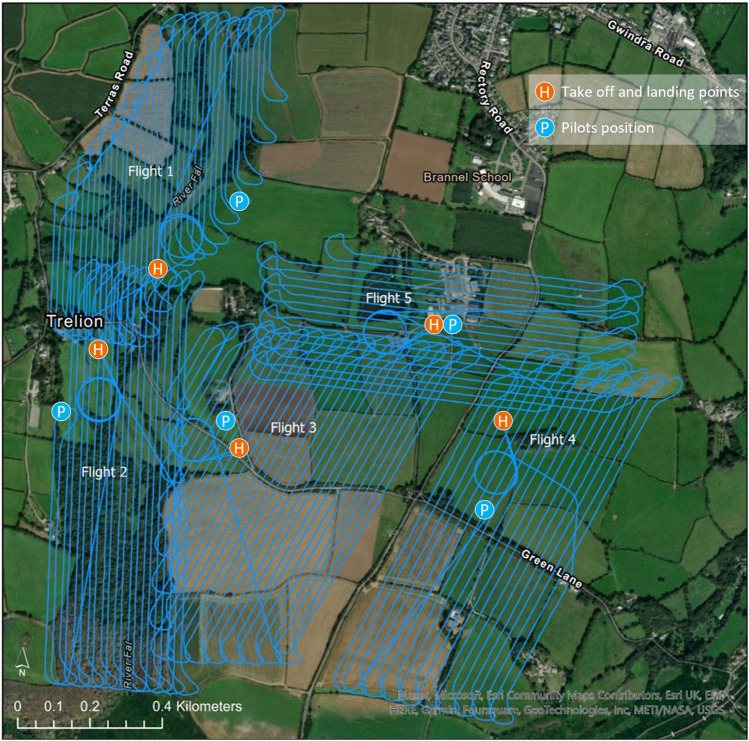
Map of the five flights completed around South Terras by the WingtraOne. Flight line spacing of 20 m was used for each flight. All flights were conducted at an altitude of 70 m.

Table 2; Figure 5 show a summary of the five flights that were completed at South Terras in 2022. Due to the slightly increased payload weight exerted by the AARM system, the Wingtra was capable of flying for approximately 40 min in the conditions experienced on the day [20–30 km h^−1^ wind speed and intermittent light rain (Newquay Airport Weather Station, 2022)]. Due to the undulating and densely vegetated terrain of South Terras, five different surveys were required to cover the extended area around the mine in order to retain a consistent visual line of sight on the UAV at all times. Should the terrain have been flatter, giving better extended visual line of sight, fewer flights would have been required. Figures 4, 5 show a small B-Road that was overflown in flights 3 and 4. Spotters, who maintained radio contact with the pilot and copilot, were tasked to intercept any oncoming vehicles during the surveys and recommend them to momentarily wait for the UAV to overfly the area if the aircraft was in the vicinity at the time. This process was included in the pre-flight risk assessment procedure and discussed during the flight briefing. Due to the operational velocity (15 m s^−1^) and altitude of the survey (70 m), it was evaluated that there was an extremely low risk to the oncoming vehicles from the UAV. Despite the operational provision for this situation, no vehicles were encountered on this road while the UAV was in flight.

**TABLE 2:** Summary of flights completed around South Terras by the WingtraOne. The locations of the five flights can be seen in Figure 5 Flight line spacing of 20 m was used for each flight. All flights were conducted at an altitude of 70 m above the take-off location and at a programmed velocity of 15 m s^−1^.

Flight number	Location	Flight length	Duration	Area
		km	min	km^2^
1	North of mine	29.16	32.33	0.2441
2	South of mine	26.93	29.78	0.3440
3	East of mine	30.08	32.93	0.4304
4	East of crow hill road	23.54	25.98	0.3923
5	Resugga farm	21.48	23.80	0.3082

A flight line spacing of 20 m was used for each of the five surveys as this allowed for good aerial coverage within a single flight battery but maintained enough overlap between flight lines that a good spatial distribution was achieved, as seen in Table 2; Figure 5. Figure 5 shows the take off and landing points of the UAV relative to the pilot in the five surveys. In surveys 1, 2,3 and 4, the pilot position and aircraft take-off location were not adjacent in order to maintain VLOS with the aircraft at all times throughout the planned route. All flights were conducted at a programmed velocity of 15 m s^−1^. In a total flight time of approximately 2.4 h, 1.7 km^2^ of land around the mine was surveyed.

### 2.4 Data processing

While the WingtraOne aircraft offers operational advantages through its mode of flight, the limited payload capacity of the aircraft restricts the volume of the spectrometers that can be integrated into the system. The use of small volume gamma spectrometers to detect and map NORM or TENORM presents challenges to analyse due to the relatively low detection efficiency of these detectors for gamma rays in excess of 1.4 MeV Lowdon et al. (2019). This characteristic often leads to poor spectral statistics in the resultant data sets, with this effect being observed within this study. Of the two spectrometers contained within the Wingtra-AARM, only the Hamamatsu CsI(Tl) unit collected spectra with sufficient statistics to allow for analysis to be completed across certain parts of the area. The smaller (1 cm^3^) CZT spectrometer was included in the payload for mapping over and around high-dose nuclear sites and facilities where the CsI spectrometer would become signal saturated.

The radiation mapping payload within the aircraft collected gamma spectra at a rate of 10 Hz, which were integrated into larger time intervals of 1, 2, and 5 Hz in post-processing. While 1 Hz is generally the preferred time interval to minimise the lateral distance associated with each measurement point, the larger time intervals were processed to provide data sets with improved statistics at every measurement point. Each spectrum in the resultant data sets was normalised for the collection time and analysed to extract the peak intensities from ^40^K, from the single peak at 1.46 MeV, and ^238^U/^226^Ra, through the 0.609, 1.12 and 1.764 MeV peaks associated with the decay of ^214^Bi. Gamma intensity information related to ^232^Th, commonly extracted from the 2.614 MeV peak associated with the decay of ^208^Tl, was not attempted due to the lack of identifiable peaks within any of the collected data sets across all time intervals.

Peak intensities were calculated by defining a series of spectral windows focused over the energies of interest, informed by the energy resolution of the Hamamatsu unit at the corresponding photon energy and determined through inspection of laboratory data. All spectra were initially smoothed using a Savitzky-Golay filter (Savitzky and Golay, 1964; Yule, 1967), for which a third-order polynomial and a smoothing window of 11 channels was used. The full energy peak intensity was determined from the spectrum by removing contributions from scattered photons and calculating the area of the remaining peak through two methods; through creating a Poisson distributed data set using the corrected peak window total and through fitting a Gaussian curve to the corrected window peak. Both methods were employed to ensure that peak values could be extracted when the photon counts were sufficiently intense to approximate to Gaussian statistics and when values were below this level (i.e., when Gaussian fitting was poor or when it failed). Contributions from scattered photons were evaluated through fitting a linear baseline across the edges of the peak window, using end-point averages over 15 channels to determine the intensity values for this baseline either side of the window. Uncertainty on the extracted peak intensities was calculated using standard error propagation techniques for independent variables, as explained with reference to gamma radiation within (Minty et al., 1997; Tsoulfanidis and Landsberger, 2021).

Surveys conducted with occupied aircraft often reduce the elemental count rates to ^40^K, ^238^U and ^232^Th radioelement concentrations using a sensitivity coefficient following altitude correction and levelling (Minty et al., 1997; Erdi-Krausz et al., 2003). As the intended application for the Wingtra-AARM is in mapping hazard in post-disaster or post-industrial environments, a reduction to radioelement concentrations was not performed. Instead, the spectral intensities were converted to dose rates, as this better describes the hazard to biological life. The conversion of whole spectrum and specific peak intensity dose rates was accomplished using a dose conversion function derived from MCNP5 calculations of planar environmental source conditions as detailed in (Tsuda and Tsutsumi, 2012; Tsuda et al., 2018) and shown inFigure 6.

**FIGURE 6:**
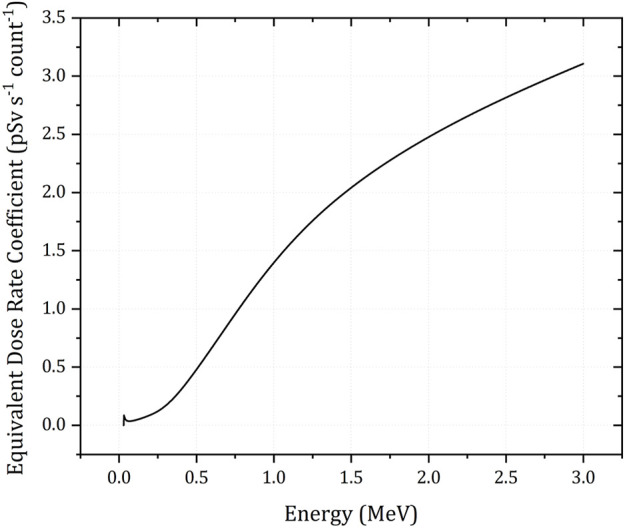
Dose conversion function for the Hamamatsu C12137-01 spectrometer in the energy range 0–3 MeV under planar environmental source conditions. Data from (Tsuda et al., 2018).

Following the conversion to dose rates, the take-off and landing phases were removed from the data and the five flights were combined into a single data set. Each measurement was then corrected for the variance in altitude above ground level (AGL) using the height recorded by the LiDAR unit on board. The altitude correction was performed using an Ei_2_ (*μ* h) correction function [see (Erdi-Krausz et al., 2003)] using linear attenuation coefficient values for standard temperature and pressure (STP) air at the corresponding photon energies obtained from National Institute of Standards and Technologies XCOM database (Berger, 2010). For the full spectrum dose, the linear attenuation coefficient was obtained from a previous survey (Connor et al., 2020). Temperature, humidity and altitude values recorded by the Wingtra-AARM during the survey were converted to STP altitudes using the method outlined in (Minty et al., 1997; Erdi-Krausz et al., 2003), with the dose rate values corrected to 1 m AGL. No attempts were made within the processing to control for radon and an infinite planar geometry was assumed throughout the survey. Visualisation of the processed data was conducted within ArcGIS Pro, with interpolation of the radiometric data achieved through the use of the Empirical Bayesian Kriging tool (ESRI ArcGis, 2022).

## 3 Results

**FIGURE 7:**
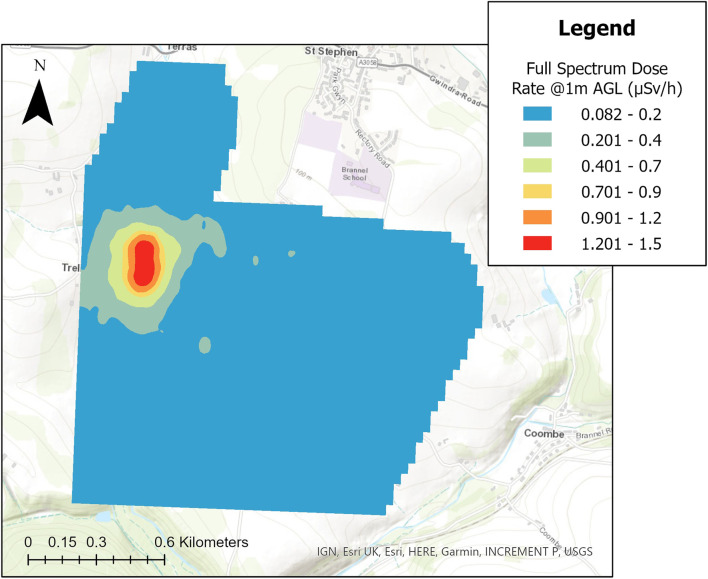
Interpolated map of the full spectrum dose rate, sampled at 2 Hz, across the survey region covered by the 5 flights.

**FIGURE 8:**
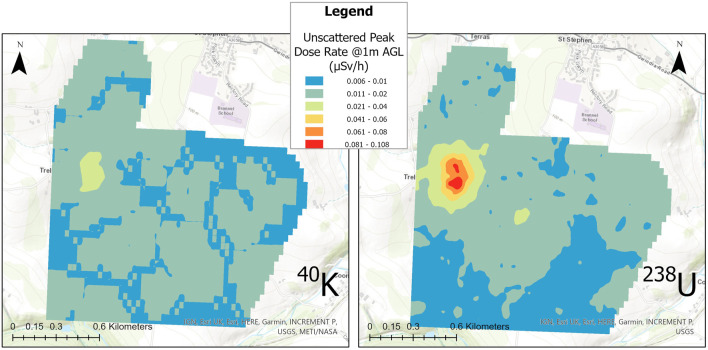
Interpolated map of the unscattered equivalent dose rates from the windowed 2 Hz spectral data for ^40^K and equivalent ^238^U. The dose rate presented here for the ^238^U window is formed from a sum of the windows centred at 609, 1,120 and 1764 keV.

The dose rate at 1 m above ground level (AGL) calculated for the whole spectrum and the ^40^K and ^238^U spectral windows are presented in Figures 7, 8, respectively. The full spectrum dose rate mapped across the area varies from 0.082 to 1.5 µSv h^−1^, with the highest dose rates being presented in the expected area surrounding the South Terras mining site. From the VTOL fixed-wing data, the peak rate within the South Terras hot spot region is between 11 and 23 times the background of the surrounding area. Thus, the hot spot completely dominates the radioactivity distribution throughout the area.

Given the limited volumes of the detector systems within the Wingtra-AARM, the full spectrum dose represents the most statistically confident method of mapping the variability across the area due to the larger amount of photons detected over using windowed intensities. However, it does not delineate the radionuclides responsible for any increases in radioactivity above background levels. The windowed dose rates presented in Figure 8 show a representation of how the variability of ^40^K and components of the ^238^U/^226^Ra decay chain affects the distribution of dose rate throughout the area. The presented dose rate for ^238^U is calculated from the summed dose rates from the ^214^Bi peaks at 609, 1,120 and 1764 keV. The windowing of the spectra to extract only the unscattered photons that have fully interacted with the detector understandably leads to a drop in the magnitude of the measured dose rate relative to the measured whole spectrum dose, as the scattered photons that form much of the spectral shape are ignored. Despite this, it can be seen that the variation across the surveyed area is mostly the product of varying uranium/radium content, with the peak dose rate from ^238^U decay chain members being at least twice as high as that from ^40^K. While dominated by uranium/radium content, definite elevations of the ^40^K intensity are observed within the South Terras hot spot, likely produced from the colocation of potassium and uranium/radium within the ore and gangue material being brought up to the surface and stored on the South Terras site. Given the limited footprint of the hot spot, the general radiological background of the surrounding area, and the industrial activity occurring in this area in the past, it is extremely unlikely that the concentration of potassium alongside the uranium/radium contamination is a coincidence produced from natural variation in the underlying geology.

A total of 4,309 points were used in the interpolation of Figure 7, separated at an effective inline point spacing of 30 m, which is calculated using the intended flight velocity of 15 m s^−1^ and sampling time of 2 Hz. In many cases, the cross line point spacing (20 m) is lower than the inline point spacing at this time sampling interval, which is not typical of these types of surveys. Considering the flight statistics, an interpolation cell size of 30 m was used, meaning that all cells likely contained either one or two points as a result. A mean of the values was used when more than one point was sampled within a single cell. With a total surveyed area of approximately 2.52 km^2^, the point density of the total data set is 1709 points/km^2^.

**FIGURE 9:**
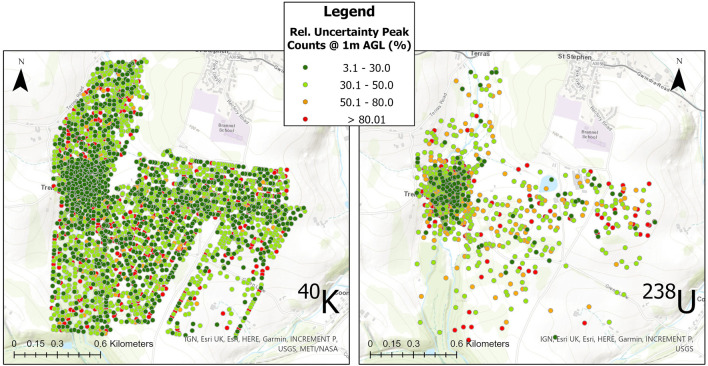
Relative Uncertainty plot for the extracted peak intensities for ^40^K and equivalent ^238^U. Measurement points with insufficient peak intensities to be discernible within the spectrum are removed. The points shown in each plot were used to produce the interpolated maps in Figure 8.

Despite presenting as an obvious hot spot within all the maps presented in this work, the overall level of radioactivity detected and mapped by the Wingtra-AARM at the survey altitude of 70 m AGL was still low, never exceeding 1.5 µSv h^−1^ (Figure 7). As a result, there was a high variability in the uncertainty of the estimated peak intensities calculated for 1 m AGL due to the low number of counts recorded by the detectors across much of the survey area. The variation in the magnitude of relative uncertainty, shown for the altitude corrected peak window intensities in Figure 9, ranged from a minimum of 3.1% to more than 400% in some cases. Significant portions of the measurements collected during the survey exhibited greater than 80% relative uncertainty (or could not be extracted due to insufficient peak counts) once processed, indicating that the radioactivity for much of the area is likely below the minimum detectable intensity at the 95% confidence level. The range exhibited by the measurements only covers those for which peak intensity values could be extracted within the bounds of the spectral windows. In the case of the uranium/radium dose, only measurements for which values for all three peak windows (609, 1,120 and 1764 keV) could be numerically determined within the spectrum are presented.

The specific potassium and uranium/radium dose rate maps presented in Figure 8 were produced from the measurement locations presented for each radioelement in Figure 9, which are sparser than the total data set used to produce the full spectrum dose rate map presented in Figure 7. While it is necessary to remove data points that could not be evaluated or that had too large an uncertainty to be used within the presented map, this procedure does alter the overall point density of the dose rate maps for the specific radionuclides. The potassium and uranium/radium dose rate maps in Figure 8 are still interpolated using a cell size of 30 m, but the distances between adjacent points in the data sets changes considerably with the removal of points, meaning that some interpolated cells contain no real measurement locations. The ^238^U dose rate map is affected by this significantly more than the ^40^K map, certain measurement points for the ^238^U map are isolated by more than 100 m, whereas a maximum isolation distance of 50 m is observed for ^40^K. The corresponding point density values for the uranium/radium and potassium unscattered dose rate maps are 332 points/km^2^ and 1,187 points/km^2^, respectively.

As can be seen within Figure 9, the hot spot region surrounding South Terras produces the measurements with the lowest magnitude of uncertainty in both of the windowed data sets. The higher magnitude of the dose rate resulting from the ^238^U decay chain in this area means that there is a higher certainty the data extracted from this series of spectral windows relative to the ^40^K window. However, the sparsity of points used within the interpolated map across the whole area shows that the limited volume CsI (Tl) detector is not suited or realistically capable of detecting variation across the lower activity regions surrounding the hot spot at the 70 m survey altitude. Although to a lesser extent, the same is true of the ^40^K window. The uncertainty calculated for measurements not within the hot spot region is consistently higher than 30% and often above 80%. Despite the limited performance of the small volume detectors around large sections of this area, the processed measurements indicate that the system is capable of delineating limited footprint hot spots produced from NORM contamination from the natural background with good confidence. More detailed spectral analysis is also shown to be possible, allowing for improved interpretation of the data even when collected at relatively elevated altitudes of 70 m AGL.

## 4 Discussion

The results from both the ^40^K and ^238^U analysis suggest that the Wingtra-AARM system is capable of detecting and localising the South Terras mine radiation hot spot, which is associated with the presence of notable NORM contamination from the previous mining activity on site.

**FIGURE 10:**
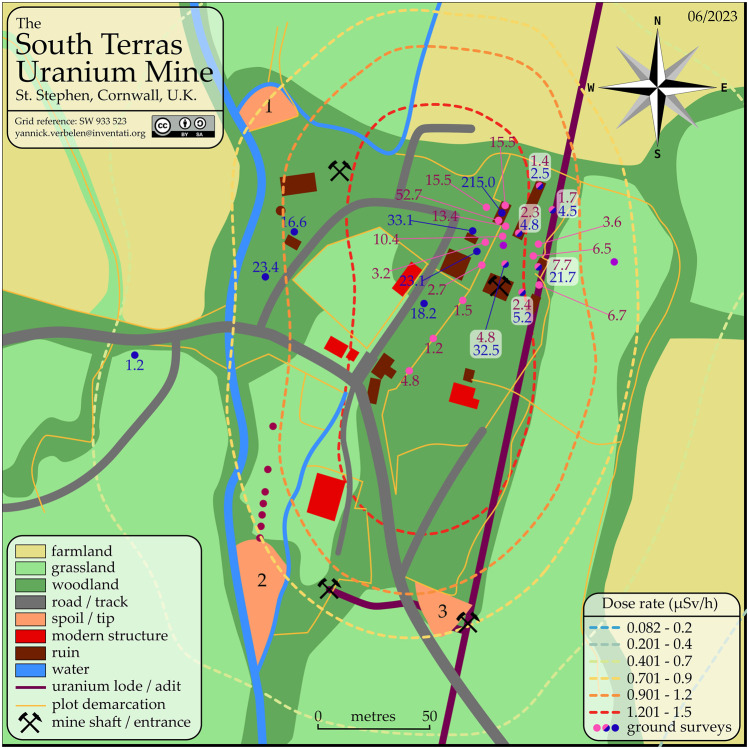
South Terras site map with plotted dose rates. The dotted contours outline the full spectrum dose rate determined from this UAV survey, representing a zoomed-in version Figure 7. The ground surveys are are plotted as coloured markers. Locations marked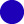 denote measurements reported by Martin et al. (2015) in *μ*Sv/h, which are presumed contact dose rates. Locations marked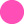 denote measurements made in this survey in *μ*Sv/h at a height of 1.0 ± 0.2 m above the surface. Locations marked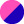 denote measurement points in common between Martin et al. (2015) and this survey, and both measurements are shown for each in their respective colours. Locations marked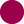 represent 7 sample points reported by Foulkes et al. (2017). Locations marked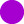 represent 2 sample points reported by Rosas-Moreno et al. (2023). Map available in vector format as open data.

An overview of ground dose rates recorded in this and previous studies are summarised in Figure 10. The ground dose rates recorded for this study in February 2023 are presented with pink markers. These were measured at an average height above ground of 1.0 m ± 0.2 m during a ground survey with a Radia code 101 portable scintillator detector, which has a sensitive volume of 1 cm^3^ CsI(Tl) crystal. Also shown are contact dose rates made by Martin et al. (2015), presented with blue markers in the same figure. Further sampling locations from studies conducted by Foulkes et al. (2017) and Rosas-Moreno et al. (2023), are also marked in on the map in Figure 10, but no comparable dose rates were recorded. These sample locations are plotted as burgundy and purple markers respectively.

The airborne dose rates collected from the Wingtra-AARM system displayed in Figure 7 show a greatly reduced total dose rate compared to those of the ground dose rates displayed in Figure 10 and relatively poor individual correspondence between these featured measurements. This is expected given the extreme modality differences between the surveys. Operating at elevated altitudes acts to average out highly heterogeneous radiological distributions (like those observed here) within airborne maps and is a well-documented phenomenon. The enlarged sampling footprint per measurement *versus* a ground-based detector system is the largest contributor to this issue (van der Veeke et al., 2021a). Residual errors could be calculated from the three different height positions we have available. However, point to point correspondence between the dose rate acquired between the ground dosimeter and UAV at 70 m will always be poor in heterogeneous situations, which based on the values from Figure 10, we can see is the case. Notwithstanding the inconsistencies, the morphology and location of the hot spot between this study and that published within Martin et al. (2015) are very similar and the Wingtra-AARM system was able to identify the presence of the hot spot and determine that it was dominated by uranium/radium series contamination with much lower levels of elevation of the ^40^K signature. These findings are in agreement with existing knowledge of the site. Much of the area surrounding the major hot spots of the site shows limited variation in the dose rate mapped by the Wingtra-AARM system, suggesting that radiation levels are much closer to the local background levels. It is envisaged that the Wingta-AARM system would have to be operating much closer to the surface to more discernibly map variation within this region.

The height above ground maintained for the radiometric surveys was approximately 70 m which was limited by the Wingtra software. Ideally, the survey height would have been 40–50 m, with a comfortable (15 m) clearance above terrain obstacles for the entire survey area. Furthermore, an improvement to the VTOL UAV would be to also allow terrain following to ensure the radiometric measurements could be safely recorded at the lowest altitudes. Implementing this feature within future bespoke aircraft systems forms part of the anticipated future work targets following this study.

Additional processing methods would allow for “hot” emitters on the ground to be more accurately delineated. Arithmetic or other reconstruction techniques employing iterative inversion methods (Minty and Brodie, 2016; Vetter et al., 2018; Connor et al., 2022) are exciting advancements to standard procedures that could be implemented to improve the results of this survey. Specifically, it would be possible to more precisely localise the South Terras radiation anomaly as well as provide confirmation of any other more moderately elevated zones of radioactivity determined in the area. Furthermore, much finer variances in dose rates could be obtained by implementing a full spectrum approach to mapping radionuclides (van der Veeke et al., 2021b). Using this approach allows for scattered photons to be included in the dose estimate for uranium and potassium, which are currently ignored within this survey. Implementing this method requires the calculation of unit spectra for the detectors within a payload, which is usually achieved through Monte Carlo software packages. Augmenting the current survey’s results with this processing method forms a second future work goal for the continuing project.

**FIGURE 11:**
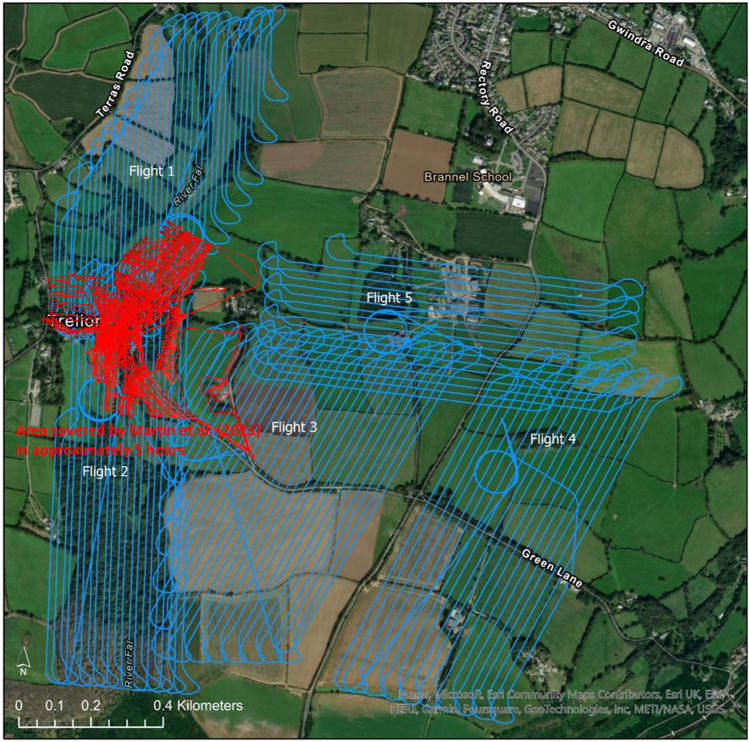
Comparison of the area covered by this study and those by Martin et al. (2015). The Wingtra covered approximately 15× the area, in half the time taken to survey the areas outlined in red by Martin et al. (2015).

Figure 11; Table 3 show the comparison in the area covered around the South Terras area, by this study and those by Martin et al. (2015) which used a multi-rotor UAV. The fixed-wing VTOL system used in this study successfully covered approximately 15× the area, in half the time taken to survey the areas outlined in red by Martin et al. (2015). Table 3 also shows how the fixed-wing VTOL system in this study compares to other conventional uncrewed fixed-wing radiation studies in terms of their flight and survey parameters, as well as the means of detection. It demonstrates the technical compromise between conventional fixed-wing and multi-rotor UAVs. The fixed-wing VTOL’s ability to fly faster and more aerodynamically than multi-rotors means that larger areas of land can be surveyed compared to those using a multi-rotor. Additionally, the VTOL removes the operational constraints such as the need for a runway and or parachute landing exhibited by fixed-wing UAVs. One benefit to the use of a multi-rotor is that its take-off and landing positions can be different. The Wingtra’s take-off and landing points are only flexible at the point of conception and cannot be altered once the survey has been commenced, these were decided to maintain VLOS at all times, as discussed in Section 2.3. If this feature is necessary the use of a different fixed-wing VTOL system with more extensive flight planning capabilities could be used.

**TABLE 3:** Comparison of different UAV-based surveys in terms of their flight and survey parameters, as well as the detection method. Error is stated in the same means it was presented in the study. Unc = Uncertainty, Std Dev = Standard Deviation and RMSE = Root Mean Squared Error. Field where parameters were omitted from respective studies were left blank.

		Flight parameters				Survey parameters			Detector			Reported data uncertainty or error
		Altitude (m)	Velocity (km/h)	Flight mode	Take-off and Landing Mechanism	Duration (h)	Area (km^2^)	Type	Volume (cm^3^)	Material	Target Radionculide(s)	
**This study**	VTOL	70	55	Auto	VTOL	2.4	1.7	Radiometric	36.1	CsI	NORM	Unc 3– > 80%
Connor et al. (2020); Connor (2021)	Uncrewed Fixed- wing	40–60	50–65	Auto	Hand launched + parachute landing	9.3	14.8	Radiometric	2 × 32.8	2 × CsI	^137^Cs	Unc 8.4%–83.7%
Geelen et al. (2022)		209–311	68–79	Auto	Catapult launched + parachute landing			Radiometric	285	CsI	^40^K	Unc 4.6%–8.2%
Pöllänen et al. (2009)		50–100	58–68	Manual	Unspecified			Radiometric	5	CsI	^137^Cs, ^192^Ir	Std Dev 2.8–5.5
Martin et al. (2015)	Multi-rotor	5–15	5.4	Manual	VTOL	5	0.12	Radiometric	1	CTZ	NORM	

A further limitation imposed by the WingtraOne platform was the 800 g maximum allowable payload weight. This limited the size and weight of the radiation detection payload, whereas ideally a CsI crystal of a larger volume would have been used to improve the gamma detection efficiency at higher photon energies. While the Hamamatsu CsI detector used in this study was able to measure gamma photons up to 3 MeV, the detection efficiency, and by extension the spectral statistics, would have been improved by utilising a larger detection crystal. This could only be achieved by moving to a larger VTOL system with a greater carrying capacity.

It should be highlighted that this variant of the Imitec AARM radiation mapping payload was designed to meet the weight requirements of the Wingtra platform but was also tailored for use over nuclear accident zones such as Chornobyl or Fukushima, where the range in gamma dose is greatly elevated in comparison to the area studied in this work. The system was not designed with NORM mapping in mind, but has demonstrated its applicability, albeit non-optimised. Other scintillator materials such as LYSO, BGO, LaBr_3_ and or CeBr_3_ are known to be better at detecting higher energy gamma photons, such as those emitted by NORM, per unit volume Lowdon et al. (2019). Looking into the utilisation of these more advanced but expensive detector materials may further improve the resolution of the data recorded by this system. Since the Wingtra-AARM system is demonstrably capable of detecting and discerning NORM radionuclides over multi-kilometre areas, further work should be conducted on the applications of this for the prospecting of valuable natural resources.

Despite a camera not being used, the internal software associated with the camera triggering speed meant that when planning and carrying out flights the Wingtra companion app will not allow for flights with an altitude of less than 70 m. Ideally, radiation surveys should be carried out as low as safely possible, which in the case of a fixed-wing UAV is approximately 40 m depending on the terrain. This could be completed with the use of an alternative VTOL fixed-wing UAV system, which may also come with the added benefit of longer flight times, allowing for an even greater areal coverage than those demonstrated in this study. The combination of COTS, like that demonstrated here, is of particular importance for many operators. However, such combinations are often associated with more rapid deployment times and reduced maintenance times. They also open up the capability to less experienced users, allowing operators to develop a mapping capability more easily.

Finally, fixed-wing VTOLs offer a great technical compromise between conventional fixed-wing and multi-rotor UAVs. Their ability to fly faster and more efficiently than multi-rotors, while not requiring runways or risky modes of landing like typical fixed-wing variants means that large areas of land can be surveyed without the operational constraints or risk faced by surveys like that of Connor et al. (2020). The VTOL capability also implies the possibility, in the future, of bringing the aircraft into a low altitude vertical hover over any determined radiation anomalies to record enhanced radiometric data, with improved counting statistics, to make spectral identification and fingerprinting of the responsible gamma sources.

## 5 Conclusion and future work

To our understanding, this study demonstrates the world’s first fixed-wing vertical take-off and landing (VTOL) UAV airborne radiation mapping survey, which was undertaken at the South Terras legacy uranium mine, Cornwall, England, United Kingdom. The survey successfully detected and localised the radiation anomaly formed by the South Terras uranium mine and refinery from a survey altitude of 70 m and above with the use of the Wingtra One and bespoke AARM radiation mapping unit. Both ^40^K and ^238^U radionuclides were detected in varying concentrations across the site suggesting that it is possible to detect and discern TENORM on a NORM background. Previous studies that have assessed the site found generally comparable levels of radioactivity, suggesting a good degree of accuracy to the radiation mapping capabilities of the Wingtra-AARM system.

A key operational advancement provided by the Wingtra-AARM fixed-wing VTOL UAV radiation mapping system was the capability to rapid survey the South Terras mine and its surrounding area. The system covered 15× the area compared to previous studies, in under half the time. The trade-off between increased area coverage exhibited by the Wingtra is observed in the reduced resolution of the data that is recorded at the Wingtra’s current minimum flight altitude of 70 m. This is compared to those of a multi-rotor drone survey which can be undertaken at as low as 1 m above the ground, depending on piloting skills.

This study has therefore proven the capabilities of using a fixed-wing VTOL UAV-based radiation mapping system to map large areas quickly yet to a good degree of resolution, making this a useful technical solution for emergency response scenarios and environmental mapping such as around nuclear facilities. Despite the Wingtra-AARM system performing its intended purpose, further improvements to the setup can be made to enhance the resolution of the radiometric data recorded. Implementation of the captured data into previously recorded digital elevation models (DEMs) alongside other airborne and or ground-based studies for the same sites can improve the accuracy of on-the-ground dose calculations further, ultimately increasing confidence in the localisation of hot spots.

## Data Availability

The raw data supporting the conclusion of this article will be made available by the authors, without undue reservation.

## References

[B1] Berger M. (2010). Nist XCOM: Photon cross sections database. Available at: http://www.nist.gov/pml/data/xcom/index.cfm .

[B2] Cai C. Carter B. Srivastava M. Tsung J. Vahedi-Faridi J. Wiley C. (2016). “Designing a radiation sensing UAV system,” in 2016 IEEE Systems and Information Engineering Design Symposium (SIEDS), 29-29 April 2016, 165. 10.1109/SIEDS.2016.7489292

[B3] Connor D. (2021). “Evaluating the use of low-cost and lightweight unoccupied aerial systems in environmental radiation mapping,” (United Kingdom: University of Bristol). Ph.D. thesis.

[B4] Connor D. Martin P. G. Scott T. B. (2016). Airborne radiation mapping: Overview and application of current and future aerial systems. Int. J. Remote Sens. 37, 5953–5987. 10.1080/01431161.2016.1252474

[B5] Connor D. Megson-Smith D. Wood K. Mackenzie R. Connolly E. White S. (2022). Mapping of post-disaster Environments using 3D Backprojection and iterative inversion methods Optimised for limited-pixel gamma Spectrometers on unoccupied aerial systems (UAS). Tech. rep., Copernicus Meetings.

[B6] Connor D. T. Wood K. Martin P. G. Goren S. Megson-Smith D. Verbelen Y. (2020). Radiological mapping of post-disaster nuclear environments using fixed-wing unmanned aerial systems: A study from Chornobyl. Front. Robotics AI 149, 149. 10.3389/frobt.2019.00149 PMC7805860 33501164

[B7] Erdi-Krausz G. Matolin M. Minty B. Nicolet J. Reford W. Schetselaar E. (2003). Guidelines for radioelement mapping using gamma ray spectrometry data. Vienna, Austria: International Atomic Energy Agency-IAEA.

[B8] ESRI ArcGis (2022). Empirical bayesian kriging (geostatistical analyst).

[B9] Foulkes M. E. Millward G. E. Henderson S. Blake W. H. (2017). Bioaccessibility of U, Th and Pb in solid wastes and soils from an abandoned uranium mine. J. Environ. Radioact. 173, 85–96. 10.1016/j.jenvrad.2016.11.030 27979647

[B10] Geelen S. Camps J. Olyslaegers G. Ilegems G. Schroeyers W. (2022). Radiological surveillance using a fixed-wing uav platform. Remote Sens. 14, 3908. 10.3390/rs14163908

[B11] Han J. Xu Y. Di L. Chen Y. (2013). Low-cost multi-UAV technologies for contour mapping of nuclear radiation field. J. Intelligent Robotic Syst. 70, 401–410. 10.1007/s10846-012-9722-5

[B12] ICAO (2021). Technical instructions for the safe transport of dangerous goods by air. Tech. rep. Montreal, Canada: International Civil Aviation Organisation.

[B13] Kutner A. Black S. Beddow H. Almond M. (2012). “ *In-situ* survey of an abandoned uranium mine in South Terras (Cornwall) containing naturally occurring radioactive material,” in International Symposium on Environmental Radioactivity: Implications for Environmental and Human Health. 1.

[B14] Lowdon M. Martin P. G. Hubbard M. W. Taggart M. P. Connor D. T. Verbelen Y. (2019). Evaluation of scintillator detection materials for application within airborne environmental radiation monitoring. Sensors 19, 3828. 10.3390/s19183828 31487922 PMC6767284

[B15] Martin P. G. Connor D. T. Estrada N. El-Turke A. Megson-Smith D. Jones C. P. (2020). Radiological identification of near-surface mineralogical deposits using low-altitude unmanned aerial vehicle. Remote Sens. 12, 3562. 10.3390/rs12213562

[B16] Martin P. G. Payton O. D. Fardoulis J. S. Richards D. A. Scott T. B. (2015). The use of unmanned aerial systems for the mapping of legacy uranium mines. J. Environ. Radioact. 143, 135–140. 10.1016/j.jenvrad.2015.02.004 25771221

[B17] Minty B. Brodie R. (2016). The 3D inversion of airborne gamma-ray spectrometric data. Explor. Geophys. 47, 150–157. 10.1071/eg14110

[B18] Minty B. Luyendyk A. Brodie R. (1997). Calibration and data processing for airborne gamma-ray spectrometry. AGSO J. Aust. Geol. Geophys. 17, 51–62.

[B19] Newquay Airport Weather Station (2022). Newquay airport weather history 18/08/22. Tech. rep. Newquay: Airport Weather Station.

[B20] Pöllänen R. Toivonen H. Peräjärvi K. Karhunen T. Ilander T. Lehtinen J. (2009). Radiation surveillance using an unmanned aerial vehicle. Appl. Radiat. isotopes 67, 340–344. 10.1016/j.apradiso.2008.10.008 19046635

[B21] Ramsankaran R. Navinkumar P. Dashora A. Kulkarni A. V. (2021). UAV-based survey of glaciers in Himalayas: Challenges and recommendations. J. Indian Soc. Remote Sens. 49, 1171–1187. 10.1007/s12524-020-01300-7

[B22] Rosas-Moreno J. Walker C. Duffy K. Krüger C. Krüger M. Robinson C. H. (2023). Isolation and identification of arbuscular mycorrhizal fungi from an abandoned uranium mine and their role in soil-to-plant transfer of radionuclides and metals. Sci. Total Environ. 876 2023, 162781. 10.1016/j.scitotenv.2023.162781 36906011

[B23] Savitzky A. Golay M. J. (1964). Smoothing and differentiation of data by simplified least squares procedures. Anal. Chem. 36, 1627–1639. 10.1021/ac60214a047 22324618

[B24] Simon M. Copăcean L. Popescu C. Cojocariu L. (2021). 3D mapping of a village with a WingtraOne VTOL tailsitter drone using PIX4D mapper. Res. J. Agric. Sci. 53.

[B25] Simpson J. E. Holman F. Nieto H. Voelksch I. Mauder M. Klatt J. (2021). High spatial and temporal resolution energy flux mapping of different land covers using an off-the-shelf unmanned aerial system. Remote Sens. 13, 1286. 10.3390/rs13071286

[B26] Smale C. V. (1993). South Terras: Cornwall’s premier uranium and radium mines. J. R. Institution Corn. 1 (3), 304–322.

[B27] Tilon S. Nex F. Vosselman G. Sevilla de la Llave I. (2022). Towards improved unmanned aerial vehicle edge intelligence: A road infrastructure monitoring case study. Remote Sens. 14, 4008. 10.3390/rs14164008

[B28] Trpković A. Čokorilo O. Jevremović S. Marina M. (2020). Application of drones for detecting and improving the quality of road signs and markings. 19th Int. Conf. Transp. Sci. (ICTS) 2020, 8.

[B29] Tsoulfanidis N. Landsberger S. (2021). Measurement & detection of radiation. United States: CRC press

[B30] Tsuda S. Tanigaki M. Okumura R. Yoshida T. Saito K. (2018). Dependence of dose rate measurement in the environment on crystal configuration of scintillation detectors. Nippon. Genshiryoku Gakkai Wabun Ronbunshi 17, 11–17. 10.3327/taesj.j16.039

[B31] Tsuda S. Tsutsumi M. (2012). Calculation and verification of the spectrum. dose conversion operator of various CsI(Tl) scintillation counters for gamma-ray. Hoken Butsuri 47, 229a–265a. 10.5453/jhps.47.229a

[B32] Türk T. Tunalioglu N. Erdogan B. Ocalan T. Gurturk M. (2022). Accuracy assessment of uav-post-processing kinematic (PPK) and UAV-traditional (with ground control points) georeferencing methods. Environ. Monit. Assess. 194, 476–512. 10.1007/s10661-022-10170-0 35665864

[B33] van der Veeke S. Limburg J. Koomans R. Söderström M. de Waal S. van der Graaf E. (2021a). Footprint and height corrections for UAV-borne gamma-ray spectrometry studies. J. Environ. Radioact. 231, 106545. 10.1016/j.jenvrad.2021.106545 33601321

[B34] van der Veeke S. Limburg J. Koomans R. Söderström M. van der Graaf E. (2021b). Optimizing gamma-ray spectrometers for UAV-borne surveys with geophysical applications. J. Environ. Radioact. 237, 106717. 10.1016/j.jenvrad.2021.106717 34419768

[B35] Vetter K. Barnowksi R. Haefner A. Joshi T. H. Pavlovsky R. Quiter B. J. (2018). Gamma-ray imaging for nuclear security and safety: Towards 3D gamma-ray vision. Nucl. Instrum. Methods Phys. Res. Sect. A Accel. Spectrom. Detect. Assoc. Equip. 878, 159–168. 10.1016/j.nima.2017.08.040

[B36] Yule H. P. (1967). Mathematical smoothing of gamma ray spectra. Nucl. Instrum. Methods 54, 61–65. 10.1016/s0029-554x(67)80007-7

